# Combination of expert guidelines-based and machine learning-based approaches leads to superior accuracy of automated prediction of clinical effect of copy number variations

**DOI:** 10.1038/s41598-023-37352-1

**Published:** 2023-06-29

**Authors:** Tomáš Sládeček, Michaela Gažiová, Marcel Kucharík, Andrea Zaťková, Zuzana Pös, Ondrej Pös, Werner Krampl, Erika Tomková, Michaela Hýblová, Gabriel Minárik, Ján Radvánszky, Jaroslav Budiš, Tomáš Szemes

**Affiliations:** 1 Geneton Ltd., Bratislava, Slovakia; 2 Department of Molecular Biology, Faculty of Natural Sciences, Comenius University, Bratislava, Slovakia; 3 Comenius University Science Park, Bratislava, Slovakia; 4 Institute of Clinical and Translational Research, Biomedical Research Center, Slovak Academy of Sciences, Bratislava, Slovakia; 5 Medirex Group Academy NPO, Nitra, Slovakia; 6 Trisomy Ltd., Nitra, Slovakia; 7 Slovak Center of Scientific and Technical Information, Bratislava, Slovakia

**Keywords:** Computational models, Computational biology and bioinformatics, Software

## Abstract

Clinical interpretation of copy number variants (CNVs) is a complex process that requires skilled clinical professionals. General recommendations have been recently released to guide the CNV interpretation based on predefined criteria to uniform the decision process. Several semiautomatic computational methods have been proposed to recommend appropriate choices, relieving clinicians of tedious searching in vast genomic databases. We have developed and evaluated such a tool called MarCNV and tested it on CNV records collected from the ClinVar database. Alternatively, the emerging machine learning-based tools, such as the recently published ISV (Interpretation of Structural Variants), showed promising ways of even fully automated predictions using broader characterization of affected genomic elements. Such tools utilize features additional to ACMG criteria, thus providing supporting evidence and the potential to improve CNV classification. Since both approaches contribute to evaluation of CNVs clinical impact, we propose a combined solution in the form of a decision support tool based on automated ACMG guidelines (MarCNV) supplemented by a machine learning-based pathogenicity prediction (ISV) for the classification of CNVs. We provide evidence that such a combined approach is able to reduce the number of uncertain classifications and reveal potentially incorrect classifications using automated guidelines. CNV interpretation using MarCNV, ISV, and combined approach is available for non-commercial use at https://predict.genovisio.com/.

## Introduction

The broad implementation of microarrays and massive parallel sequencing (MPS) technologies leads to the unleashing of an ever-increasing genetic variability. This raises the demand for correct understanding and interpretation of the impact of the identified variants on human health, especially in clinical settings. The interpretation of genomic variants is complex and often yields ambiguous results, which may even vary between laboratories. In order to alleviate this obstacle, technical standards for the interpretation and reporting of variants have been developed by the American College of Medical Genetics and Genomics (ACMG) and the Association for Molecular Pathology (AMP). The first initiative was developed for short sequence variants^1^, as they are better understood. Although some of the basic rules for the interpretation are consistent across all types of genomic variability, these standards are not suitable for the large-scale variants, such as copy number variations (CNVs). Evaluation of CNVs’ clinical impact is really challenging since these unbalanced structural genomic aberrations may range from 50 bps up to several Mbps. CNVs typically affect multiple functional genomic elements simultaneously, leading to diverse biological roles, ranging from having no effect on common physiological traits to the development of severe genetic disorders^2^, and thus specific rules for interpretation must be applied.

At present, several approaches are available to clinicians in the decision-making process regarding interpretation of CNVs’ clinical significance^3^. In the main approach, CNVs can be classified following a recently published joint consensus recommendation of the ACMG and the Clinical Genome Resource (ClinGen) (in brief: ACMG criteria)^4^, by selecting options and respective score values for individual guideline categories^5–7^. These professional standards tend to encourage consistency and transparency of CNV evaluation across clinical laboratories. In another approach, CNV classification is based on fully automated machine learning algorithms that can go beyond the established scheme, thus having a great potential to further improve the prediction accuracy^8–10^. The CNVs’ pathogenicity effect can also be evaluated according to the aggregation of per-base single nucleotide polymorphism (SNP) pathogenicity scores within the CNV intervals^11^. One example of a tool that uses this approach is the SVscore^11^, which aggregates the SNPs’ pathogenicity scores according to CADD (Combined Annotation Dependent Depletion)^12^. Recently, another expert system for CNVs’ clinical impact classification has been presented—ABC system^13^ consisting of three-step classification: functional grading (A), clinical grading (B), and optional selection of standard variant comment based on a combined class (C). Authors propose to use it independently or as a supplement to the ACMG system.

The approach based on ACMG guidelines follows a scoring scheme divided into five basic sections: (1) Initial Assessment of Genomic Content; (2) Overlap with Established Triplosensitive (TS), Haploinsufficient (HI), or Benign Genes or Genomic Regions; (3) Evaluation of Gene Number; (4) Detailed Evaluation of Genomic Content Using Cases from Published Literature, Public Databases, and/or Internal Lab Data; and (5) Evaluation of Inheritance Patterns/Family History for Patient Being Studied^4^. In other words, it is based on the genomic content of the identified CNV, namely on evaluating whether the region covered by the given CNV also overlaps features such as protein-coding genes or elements with important functions, known HI/TS genes or known benign CNVs. Detailed evaluations of CNVs’ genomic content from the published literature, studies, and available databases are also included, together with the family history of the patient. Based on the sub-intervals of the final score, the 5-tier classification system is implemented, and the potential clinical impact of the tested CNV is classified as: benign (B) (≤ − 0.99), likely benign (LB) (from − 0.98 to − 0.90), uncertain significance (VUS) (from − 0.90 to 0.90), likely pathogenic (LP) (from 0.90 to 0.98) and pathogenic (P) (≥ 0.99).

The main purpose of the guidelines is to assist clinical laboratories in the classification and reporting of CNVs’ clinical impact, irrespective of the technology used to identify them. However, many CNVs are evaluated as VUS, thus, frequently inconclusive, and follow-up classification and validation/curation by a clinician are needed (not seldom after several years). Another inconvenience is the frequent need for a clinical expert to spend a long time browsing various data sources and publications in order to evaluate individual categories. Selection of different options and assignment of a score for each category is not immediate as the subjective judgment of the clinician may influence the score adjustment in several categories. Moreover, repeatedly experiencing the given finding may lead to a change in CNV reclassification over time. Thus, based on these criteria, different clinicians may arrive at conflicting CNV classifications.

This has driven the effort to create semi-automated tools that can partially eliminate the tedious process of annotating and assigning a certain score for CNVs by automating the search for information related to the genomic content of CNVs from various sources/databases. Examples of such classification tools that help to automate CNV annotation and classification of individual sections of ACMG criteria include AnnotSV^5^, ClassifyCNV^6^ and AutoCNV^7^. These tools slightly differ in the data sources they use, in the categories from five sections of ACMG criteria that they are able to evaluate (Table 1), and in the way they interpret the details of the ACMG criteria. Thus, the subsequent final score of CNV and the classification of its clinical impact by different tools may differ. In order to finalize the evaluation, sometimes it is still necessary to search for genomic content of the CNV in the published literature and to manually evaluate the patient’s family history.

**Table 1:**
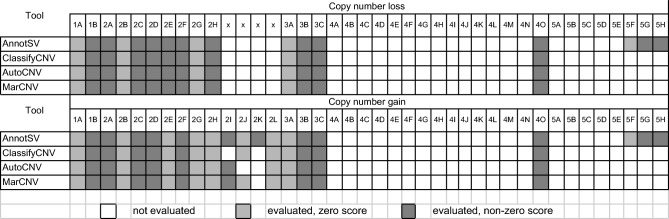
Sections/parameters of the ACMG criteria that are evaluated/not evaluated by the listed semi-automated tools for determining CNVs’ pathogenicity. The MarCNV tool was designed by us (see “Methods” section).

On the contrary, approaches predicting the functional impact of CNVs based on specific machine learning algorithms can be fully automated. Several in silico CNV pathogenicity prediction tools have been reported recently, differing in the machine learning algorithms they implement, in the usage of specific genomic elements as features for model training, and in the parameters applied for model training. One of the tools is StrVCTVRE^10^ focusing on exons overlapped by structural variants. The model was trained on CNVs collected from the ClinVar database using 17 annotations characterizing gene importance, coding region, conservation, expression, and exon structure as features implemented in a random forest classification. Another available method for pathogenicity prediction is X-CNV^8^, the model trained using the XGBoost classifier. Recently, we have developed a tool called ISV (Interpretation of Structural Variants)^9^ which predicts the probability of pathogenicity of a CNV and its likely clinical impact based on specified CNV coordinates (see the ISV subsection in “Methods” for details).

In this paper, we present an automated tool called MarCNV to evaluate mainly the first three sections of the ACMG criteria. We provide evidence that both the database choice and the parameters’ selection influence the resulting score when implemented in some sections of the ACMG criteria. Finally, we evaluated the clinical impact of the selected CNV test sets (from the ClinVar database) using MarCNV and ISV (machine learning approach), both individually or together as their combination. We show that the combined approach is able to reduce the number of uncertain classifications (VUS) and reveal the potentially incorrect classifications according to automated guidelines.

## Results

**Figure 1:**
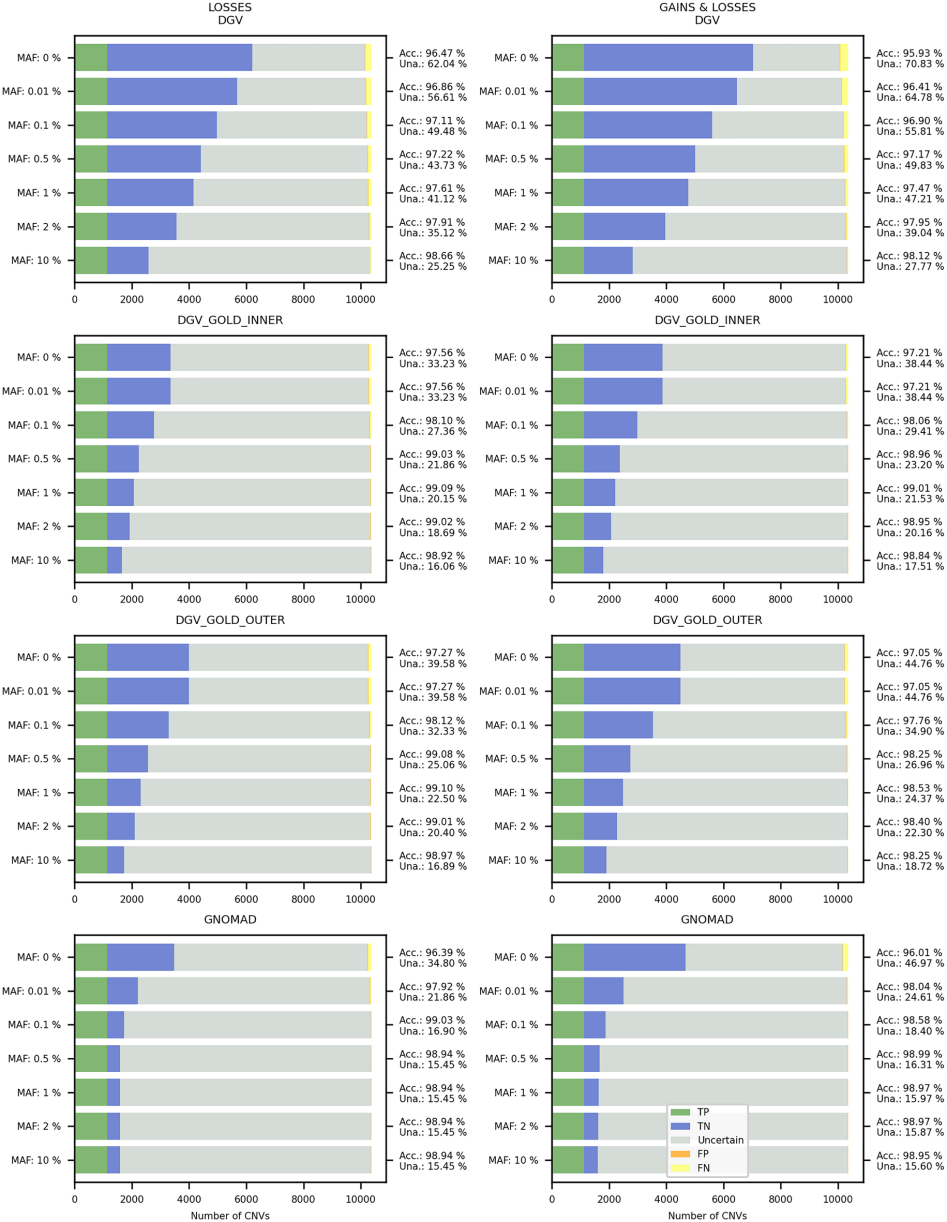
Evaluation of the option 2F for CNV losses by MarCNV using different databases of benign CNVs and their settings. The left column represents analyses performed selecting only “losses” in the search for the *established benign CNV region*, while on the right, both “gains & losses” were selected (as recommended by ACMG). GnomAD^14^ and DGV^15^ databases that collect common CNVs from large-scale sequencing studies were searched, and the results were compared. DGV database was represented in three versions, namely “DGV” that represents all CNVs in the DGV database; “DGV-GOLD” stands for “Gold Standard” and represents curated CNVs with INNER or OUTER CNV coordinates. Database search was performed at different minimum population frequency thresholds (“MAF”) set for a known/established benign variant, with the assumption that a more common CNV is less harmful. The *accuracy* (Acc) indicates the proportion of correctly evaluated CNVs, whereas *unambiguous* (Una) represents the percentage of predicted CNVs falling either into the B or P category (see Methods for details). We found the DGV-GOLD-OUTER at population frequency 0.5% to be the best. *TP* = True positive, *TN* = True negative, *FP* = False positive, *FN* = False negative, where “positive” predictions correspond with pathogenic and “negative” with benign predictions.

Here, we present MarCNV, a tool for the automated evaluation of certain sections of the ACMG criteria^4^. The MarCNV input consists of the target CNV coordinates in the format chromosome, start, end (using hg38 reference), and a definition of the CNV type (gain or loss). Subsequently, the tool evaluates complete Sections 1 and 3, almost complete Section 2, and option 4O from Section 4 of the ACMG criteria. We tested the efficacy of MarCNV using several CNV test sets from ClinVar, evaluated the influence of database parameters selection on the clinical assessment of CNVs, and found a way to improve the ACMG criteria. Figure 1 uses the concatenation of 2 datasets (*Training*, *Validation*) both of which contain CNVs smaller than 5 Mbp and deleted on one chromosome only. The remaining figures use three testing datasets: *Testing basic (deleted on one chromosome and shorter than 5 Mbp), Testing* > *5Mbp (deleted on one chromosome and larger than 5 Mbp), and Testing multiple (deleted on both chromosomes).* For more info, see the section “CNV datasets” in Methods. We have managed to join two approaches, namely classification according to the ACMG criteria using MarCNV and automated prediction by ISV, and merge them to create a combined approach (MarCNV + ISV). Moreover, we have demonstrated that this approach not only increases the accuracy of the prediction but also reduces the count of evaluation of CNVs as VUS. We have also performed several comparisons between the individual and combined approaches and compared them with the available ClassifyCNV tool. In the end, using CNVs from the clinical laboratory practice, we verified and compared evaluations performed by different approaches.

### Automated ACMG evaluation—MarCNV

MarCNV is an automated tool for evaluation of mainly the first three sections of the ACMG criteria^2^. After input of the coordinates of the target CNV in hg38 reference (chromosome, start, and end) and its character (gain or loss), this tool evaluates complete Sections 1 and 3, almost complete Section 2, and option 4O from Section 4 of the ACMG criteria.

#### The choice of a database influences the precision of CNV evaluation

We used MarCNV to evaluate some parameters and their effect on the result of the CNV evaluation process. We observed that some sections of ACMG evaluation are very sensitive to the choice of database and its preprocessing. As an example, we evaluated the option 2F for CNV losses (2F. *Completely contained within an established benign CNV region*) using different benign CNV databases and filtration settings (Fig. 1). We observed that the different filtration settings worked as a trade-off parameter between the *accuracy* of the prediction and the proportion of evaluated CNVs (*unambiguous*). Both parameters varied greatly for tested databases: *accuracy* from 95.92% (GnomAD, no filtration) to 99.02% (DGV-GOLD-INNER, frequency ≥ 1%); *inclusion* from 15.71% (GnomAD, frequency ≥ 10%) to 70.96% (DVG, no filtration). As the best option, we have chosen DGV-GOLD-OUTER with a frequency of ≥ 0.5%, as it has almost the best *accuracy* while *including* almost a quarter of all CNVs (*accuracy* = 98.26%, *inclusion* = 27.28%) (Fig. 1).

Using MarCNV, the current ACMG criteria can be adjusted to test for more *accuracy* and/or *inclusion*. For the option 2F, during CNV loss classification, we demonstrated that including only benign losses in the database search does not provide any major advantage compared to the option chosen by ACMG, where both losses and gains are included. Therefore, in all the following tests we continued with the original ACMG option (Fig. 1).

**Figure 2:**
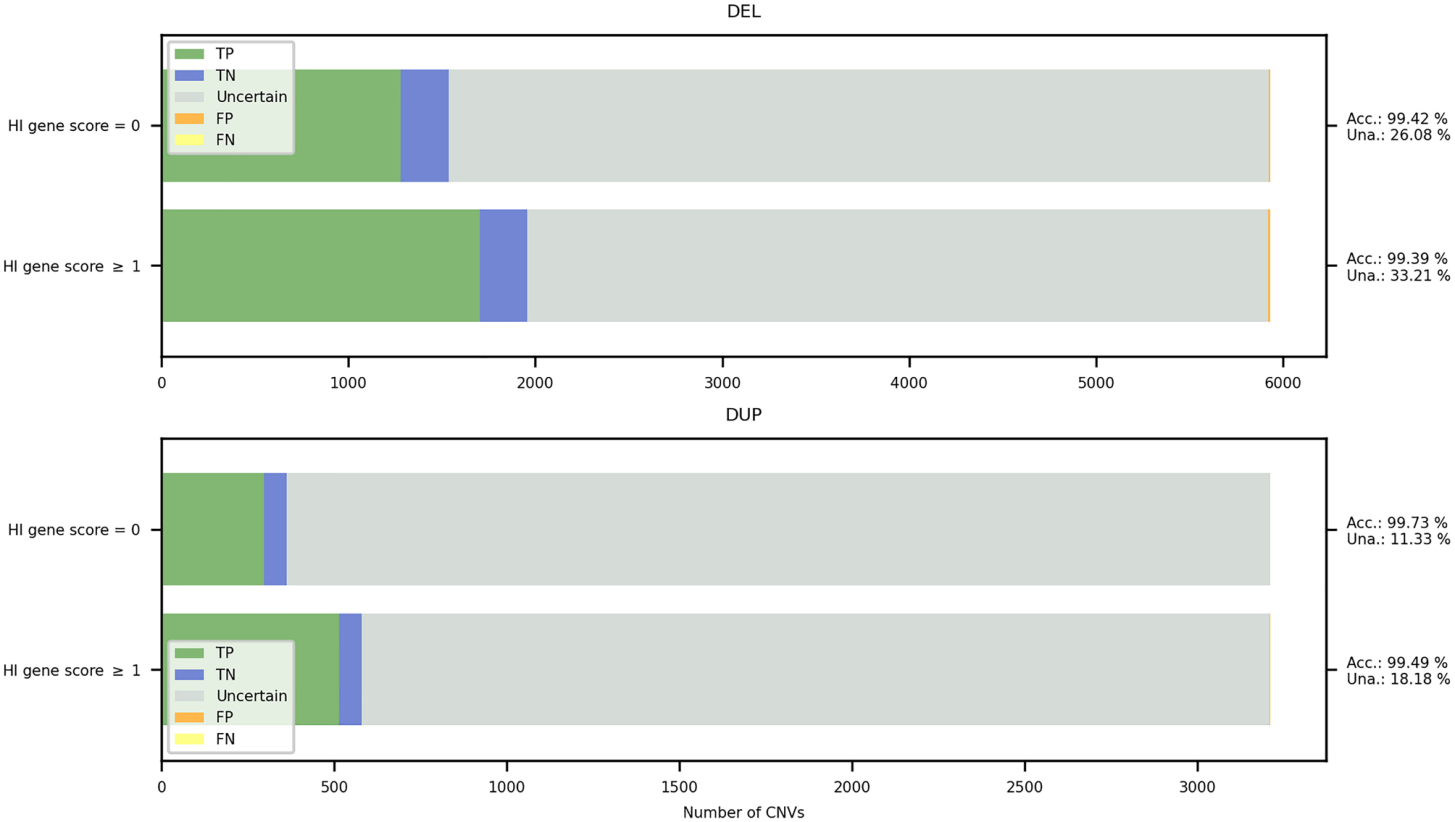
Comparison of options for HI/TS genes for evaluation of the ACMG criteria (sections 2A and 2B)—both losses (DEL, upper panel) and gains (DUP, lower panel) were analyzed. “HI(1,2,3): 0” represents an option where only HI/TS genes/regions with a score of 3 were taken into account (as in regular ACMG guidelines); “HI(1,2,3): 1” represents a search where also HI/TS genes with scores of 1 or 2 were included (together with those with a score of 3). The *accuracy* (Acc) indicates the proportion of correctly evaluated CNVs, whereas *unambiguous* (Una) represents the percentage of predicted CNVs falling either into the B or P category (see Methods for details). The DGV_GOLDOUTER database with frequency ≥ 0.5% was used for parsing benign CNVs. The x-axis represents the number of CNVs. *TP* = True positive, *TN* = True negative, *FP* = False positive, *FN* = False negative.

For options 2A and 2B (*complete and partial overlap of an established HI/TS genomic region*), we demonstrate that the inclusion of HI/TS genes with the score 2 or even 1 in the database search (in addition to those with score 3 from the ACMG settings) increased the number of predicted CNVs, while only slightly decreased the accuracy (Fig. 2).

#### Comparison of MarCNV with ClassifyCNV

**Figure 3:**
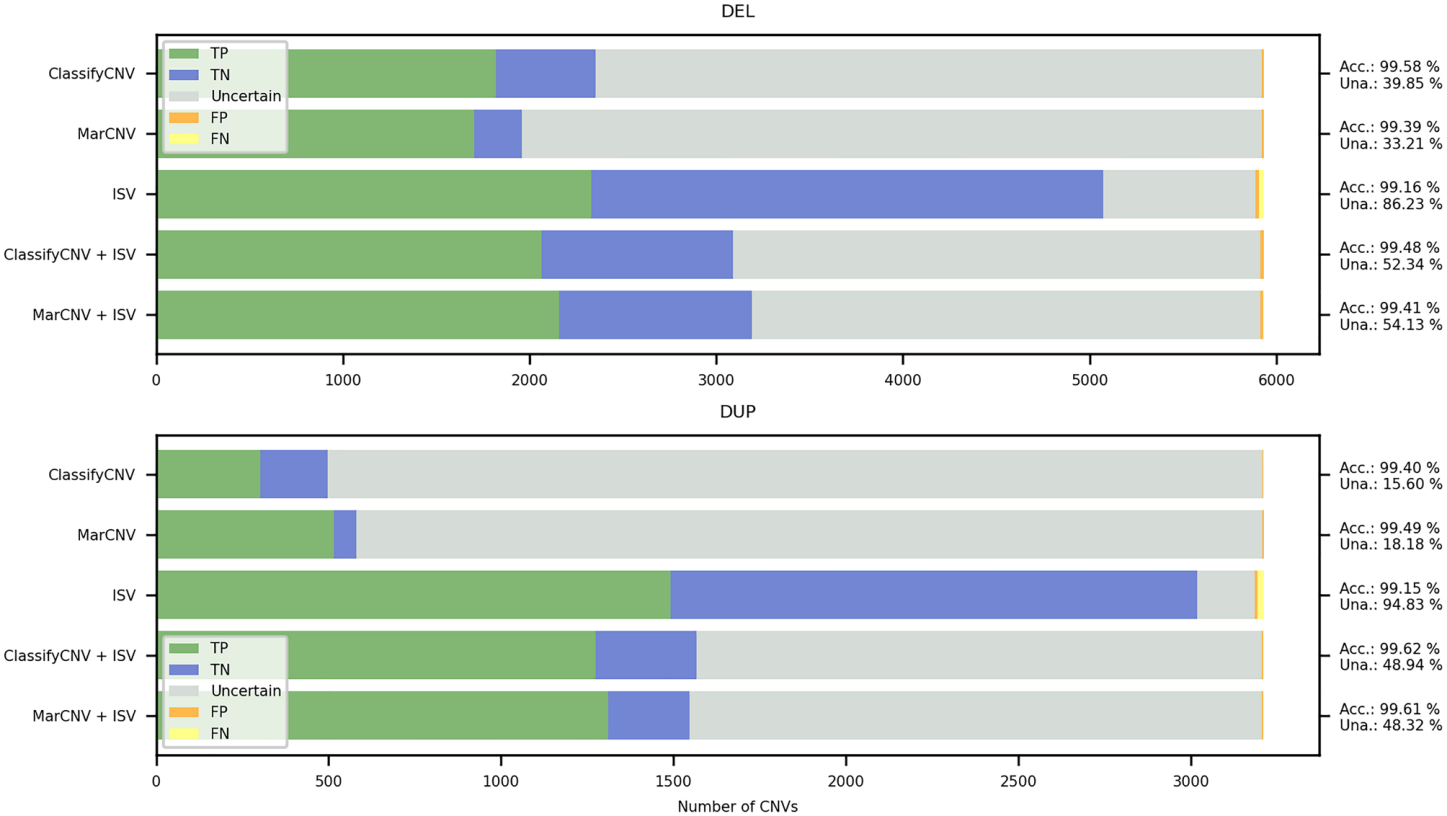
Comparison of ClassifyCNV, MarCNV, ISV, ClassifyCNV + ISV, and MarCNV + ISV methods of CNV evaluation—both losses (DEL, upper panel) and gains (DUP, lower panel) were analyzed. The *accuracy* (Acc) indicates the proportion of correctly evaluated CNVs, whereas *unambiguous* (Una) represents the percentage of predicted CNVs falling either into the B or P category (see Methods for details). The DGV_GOLD_OUTER database with a frequency of ≥ 0.5% was used for parsing benign CNVs. The x-axis represents the number of CNVs. *TP* = True positive, *TN* = True negative, *FP* = False positive, *FN* = False negative, where “positive” predictions correspond with pathogenic and “negative” with benign predictions We decided to keep the ISV ratio to 1 for the model to not rely solely on ISV if the ACMG scheme is unable to classify the CNV and outputs a score of 0.

The main difference between MarCNV and ClassifyCNV is the use of different databases for the annotation of tested CNVs (Table 2). The ACMG criteria are rather strict in evaluation, however, there are still minor differences between MarCNV and ClassifyCNV in their interpretation. For example, to evaluate the overlap with haploinsufficient genes (Sections 2C–2E for loss), the ACMG guidelines do not exactly specify which transcript of the gene should be considered. MarCNV evaluates all transcripts in the database (and subsequently reports the most severe score), while ClassifyCNV evaluates only the most established transcript. MarCNV cannot distinguish between options 2J (*patient’s phenotype is either inconsistent with what is expected for LOF of established HI gene OR unknown*) and 2K (*patient’s phenotype is highly specific and consistent with what is expected for LOF of established HI gene*) for copy number loss due to unknown patient phenotype, which is a necessary piece of data enabling the decision between these two options. However, it can evaluate option 4O (*overlap with common population variation*) for both copy number loss and gains. The *accuracy* and *inclusion* of MarCNV and ClassifyCNV are quite comparable (see Fig. 3), while ClassifyCNV evaluated fewer CNVs as VUS when compared to MarCNV. Similarly, when comparing accuracy of those methods of the evaluation of benign CNVs (Supplementary Fig. S1) or pathogenic CNVs (Supplementary Fig. S2) it can be seen they are quite similar except for the proportion of evaluated CNVs.

**Table 2:** Set of the public databases used by MarCNV in the process of automated CNV evaluation.

	ACMG section	Database
Genes	1, 3, 2C-G, 2L for gain, 2C-2E for loss	Genecode (v37)^20^
Regulatory regions	1	RefSeq^21^
Haploinsufficient and triplosensitive genes and regions	2A-B, 2H for gain, 2C-2E for loss	ClinGen^22^
Known benign CNVs	2D-G for gain, 2F-G for loss	DGV^15^
Gene annotations	2H for loss	dbNSFP (v4.0c)^23^
Population variability	4O	GnomAD^14^

### A novel approach combining MarCNV and ISV

#### Application of the new combined approach can increase *accuracy*

We confirmed that the ACMG scoring scheme is rather strict, and the majority of CNVs are classified as VUS. On the other hand, the machine-learning tool ISV is able to classify many more CNVs, however, it does it at the cost of producing more false predictions. We show in Fig. 3 that the combined use of these methods yields a superior predictor, with higher *accuracy* than the raw ISV model, and more predicted CNVs (*unambiguous*) than the ACMG scheme alone (see also Supplementary Fig. S3 for direct comparison of ClinVar classification of CNVs from the test set and their interpretation using our combined approach). Correspondingly, the accuracy comparisons of methods were made for benign CNVs (Supplementary Fig. S1) and pathogenic CNVs (Supplementary Fig. S2). The pairwise comparison of methods using the McNemar-Bowker test with k = 3 (there are three classification classes—Benign, Uncertain, and Pathogenic) showed that the methods behave quite differently from each other. The comparison between MarCNV + ISV and ClassifyCNV + ISV yielded the lowest values of the test statistic, both for copy number loss (B = 39.86) and copy number gain (B = 46.03) variants. Nevertheless, all of the comparisons yielded a p_value less than 10^−8^ (Supplementary Table S1).

**Figure 4:**
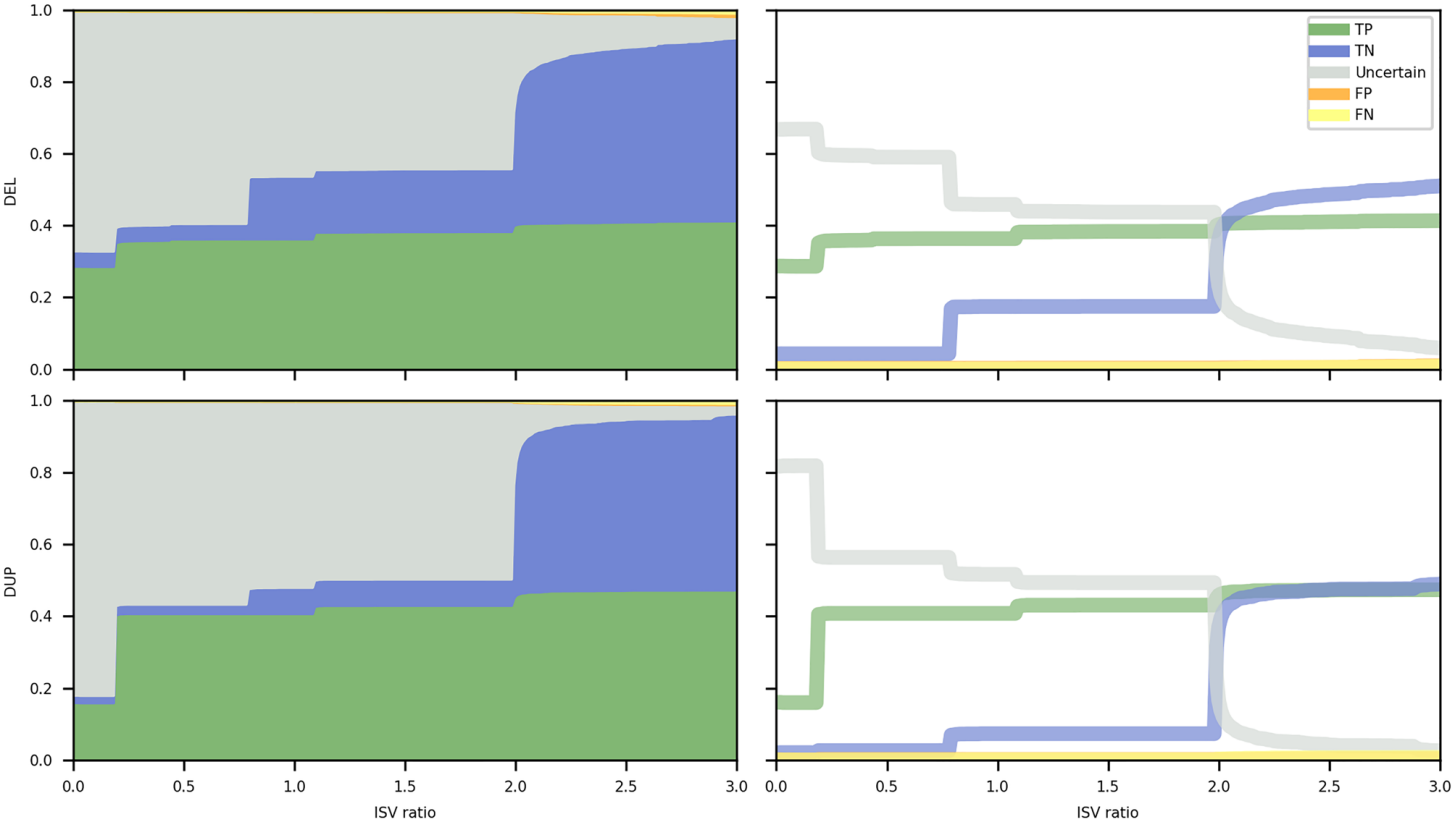
ISV ratio in the combined approach for evaluation of CNV losses (DEL, upper panel) and gains (DUP, lower panel). The left column represents relative counts of True, Uncertain, and False predictions, while the right one contains the same information plotted as a line chart individually for each category in order to facilitate comparison of the behavior of individual categories with increasing ISV ratio. A sudden decrease in the count of *uncertain predictions* (gray area) is visible, especially for ISV ratios at *r* ≃ 0.19 and *r* ≃ 1.99. The Y-axis represents the area (left) or proportion (right) of each individual category at a given ISV ratio (x-axis). Color code for individual categories is the same for both columns and is indicated in the figure (*TP* True positive, *TN* = True negative, *FP* = False positive, *FN* = False negative, where “positive” predictions correspond with pathogenic and “negative” with benign predictions).

We observed that *accuracy* of the combined approach depends on the MarCNV score and the ISV ratio (*r*) setting (see Methods), where raising *r* will increase the influence the ISV has on the final prediction. As can be seen in Fig. 4, two ISV ratios are particularly interesting: the first *r* ≃ 0.19 and the second *r* ≃ 1.99. In both these ratios, we observe a sudden drop in the count of uninformative predictions (gray area). The first drop represents CNVs with an ACMG score of 0.9, thus, they would normally be classified as LP according to the ACMG scheme. However, when ISV yields probabilities very close to 1, these CNVs will suddenly be classified as pathogenic. This sudden decrease in both ratios is more prevalent for CNV gains.

For example: If a CNV has ACMG score (MarCNV) of 0.9 and ISV probability of 0.99, then according to the model above with *r* = 0.19, we would get the final score of: 0.9 + 0.19 * (0.99–0.5) = 0.9931, which can be classified as P according to the ACMG guidelines. This is partly caused by the overconfident ISV predictions. Most CNVs usually have either very high or very low ISV probabilities of pathogenicity, and that is why the ratio *r* = 0.19 causes the increase of classifiable CNVs. The second sharp increase comes at ratio *r* = 1.99, where most CNVs with ACMG score close to 0 (VUS) became classifiable after the ISV inclusion. This stems from the fact that if ISV predicts CNV as B, it will be very confident and return very low probabilities (around 50% of the predicted B CNVs show ISV probabilities lower than 0.02). In both of these cases (0.19 and 1.99) the ratios practically first scale the probability by a factor of 2 and then by a factor of 0.095 and 0.995, respectively, which are roughly the differences between a CNV being classified as P/B from LP/LB, or as P/B from Uncertain.

A similar analysis for the ratio 0.19 and 1.99 was performed on clinical samples, which will be described below (see also Supplementary Fig. S4), where the reduction of CNVs classified as VUS was also confirmed (for r = 1.99).

#### The combined approach leads to an increased number of informative CNVs compared to ACMG

Compared to the classification following the ACMG criteria, using a combined approach, we can increase the number of informative CNVs (falling either into the B or P category). Figure 4 also shows a reduction of CNVs evaluated as *uncertain predictions* (other than B or P, gray area) when increasing the ISV ratio. When the ACMG criteria (MarCNV) with minimal ISV ratio are used, many CNVs are evaluated as *uncertain predictions*. With an increasing ISV ratio in the combined approach, a decrease in the number of CNVs evaluated as *uncertain predictions* can be observed. However, with the decreasing CNV’s evaluation as *uncertain*, there is an increase in false predictions.

On the other hand, six CNVs were found in the testing datasets, for which both MarCNV and ISV provided predictions contradicting the ClinVar labels. The CNVs (losses of chr22:18750783-25518,625, chrX:155239112-155691896, chrX:77775163-77949550, chr1:16951500-17071209, and chr1:145138148-146401981, and gain of chr2:7495123-87705899) either overlap with an established haploinsufficient gene/genomic region and/or contain known pathogenic CNVs. Therefore, MarCNV and ISV classified them as P, regardless of the B ClinVar label. Moreover, some of these CNVs have become obsolete and are no longer valid in the dbVar database. More details on conflicting predictions are provided in Supplementary Notes.

#### Verification of the combined approach on the clinical laboratory CNV classifications

We used our combined approach (MarCNV + ISV) to reanalyze 63 monoallelic CNVs evaluated previously by the clinical laboratory. The clinical interpretation (CI) was performed by a routine workflow, including public databases browsing and patient phenotype evaluation, but also considering the growing in-house/internal database, as well as the frequency of CNV in question. CNV classification workflow of the clinical laboratory was supplemented by the ACMG and ClinGen technical standards after its release by Riggs et al. in 2020. The coordinates and CI of CNVs are listed in Supplementary Table S2.

**Figure 5:**
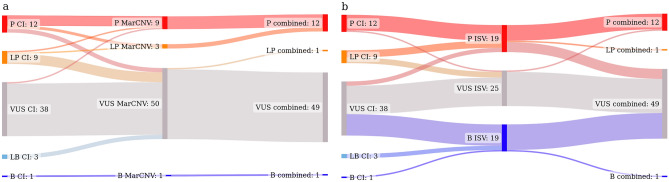
Change in the number of CNVs (obtained from the clinical laboratory) assigned to individual categories using different approaches. The middle column displays the evaluation performed by MarCNV only (panel **a**) (classification according to the ACMG criteria); or by ISV only (panel **b**) (machine learning approach). In both panels, the first column represents evaluation by clinicians (CI = Clinical Interpretation) and the third one evaluation by the combined approach (MarCNV + ISV) at ISV ratio set to *r* = 1. Line thickness also illustrates the number of CNVs in individual categories (listed in the Supplementary Table S2. *B* = Benign, *LB* = Likely benign, *VUS* = Variant of uncertain significance, *LP* = Likely pathogenic, *P* = Pathogenic. Figure was created using the SankeyMATIC tool^16^.

Nine P CNVs, one B CNV, and 37 VUS were assigned to the same category by clinicians as well as by our combined approach. Two LP CNVs, as classified by CI, were evaluated as P using the combined approach. Similarly, seven LP and three LB were classified as VUS, and one VUS as P (see Supplementary Table S2). Moreover, none of the CNVs was evaluated incorrectly—LP/P to LB/B or vice versa. The comparison of these CNV classifications using CI, MarCNV only and the combination of MarCNV + ISV is shown in Fig. 5a. We observed that evaluation according to the ACMG criteria (using MarCNV) is quite conservative (compared to CI) since most of CNVs classified as LP and LB using CI were classified as VUS by MarCNV. Adding the ISV evaluation (combined approach) resulted in a reduction of conservative CNV classification by MarCNV: three LP CNVs were changed to P and one VUS CNV was changed to LP. A similar comparison between CI, and classification performed by ISV only and MarCNV + ISV combined approach is shown in Fig. 5b. There was a match between CI and ISV evaluation for 11 P, 20 VUS, and one B CNVs. One P CNV (as by CI) was evaluated as VUS by the ISV tool, and five LP CNVs were predicted as P by the ISV tool. After adding MarCNV to the ISV evaluation the classification became more conservative since 49 CNVs were classified as VUS, while ISV alone predicted only 25 CNVs as VUS. Direct comparison of the CI with combined approach was also performed, setting the ISV ratios to 0.19 and 1.99, representing two interesting thresholds, at which a significant reduction in the classification of CNVs as VUS occurs (see also section ‘Use of a new combined approach can increase accuracy’ for more details about these ISV ratios) (Supplementary Fig. S4; Supplementary Table S2).

When we compared the results of CNVs classification between MarCNV and ISV (Supplementary Fig. S5), we observed that MarCNV evaluates more CNVs as VUS in comparison to ISV, and no CNV is classified as LB or LP by ISV. Taken together, our results indicate that both the CI and evaluation according to the ACMG criteria (using MarCNV) lead to the assignment of many CNVs to the VUS category. In comparison, the ISV prediction tool changed the predictions of 18 CNVs that clinicians classified as VUS into B (15 CNVs) or P (3 CNVs). However, this may produce some false predictions.

## Discussion

Expert-designed ACMG criteria for CNV classification represent a comprehensive scheme and are already widely used by clinicians. These ACMG and ClinGen standards for interpretation and reporting of CNVs provide a semi-quantitative point-based scoring system based on the most relevant categories of evidence^4^. Using the scoring metrics, a laboratory geneticist should assign any CNV reported in a patient to one of five main classification categories^4^. Most sections of the ACMG criteria can be analyzed by automated processes and several tools were created for this purpose, including MarCNV presented in this paper. Such computational methods recommend an appropriate selection of criteria, relieving clinicians from tedious search in vast genomic databases. We evaluated MarCNV and compared it to previously reported ClassifyCNV using several test sets of CNV records collected from the ClinVar database. This approach enabled us to confirm their comparable performance.

The ACMG criteria assess the pathogenicity of a patient’s CNV, also considering the phenotype, inheritance pattern and family history, attributes that are not always available. MarCNV is able to evaluate the sections dealing with the genomic location and population frequency of the evaluated CNV, not inevitably the clinical manifestation in the patient, and thus it works mainly for Sections 1–3 of the ACMG scheme.

We have demonstrated that the *accuracy* of a classification and the number of *unambiguous* variants vary between databases of known benign CNVs (Fig. 1). Since databases can include different sets of CNVs, the ACMG evidence-based scoring framework (e.g., Sections 2F–G for losses and 2C–2G for gains) is susceptible to the choice of database. For example, the recognition of CNV finding as 2F (*CNV is completely contained within an established benign CNV region*) and 4O (*CNV is overlapped with common population variation*) depends on the presence of its coordinates in the selected database. If the CNV is present, a given score can be negative enough to evaluate such CNV as benign. However, if not, the CNV falls into the VUS category. Moreover, considering the assumption that a more common CNV is less harmful, we tested the performance of the ACMG criteria for several CNV population frequency thresholds. The DGV database curated with MAF ≥ 0.5% and outer coordinate representation provided the best trade-off, achieving almost the highest accuracy while including almost a quarter of all CNVs (accuracy = 98.26%, inclusion = 27.28%).

We also show that the current ACMG criteria can be adjusted to allow for more *accuracy* and/or *unambiguous*, as well as improve the CNV evaluation. We found that including HI/TS genes with lower scores increased the number of predicted CNVs, while it only slightly decreased the *accuracy*. We continue testing additional improvements to the ACMG criteria and implement them to MarCNV in a way enabling the evaluation of any given CNV with “standard” or improved ACMG criteria.

The output file of the classification according to the ACMG criteria contains evaluation in five sections, also showing which of the criteria was selected as well as points acquired to contribute to the final evaluation of clinical significance. In the machine learning approach, the effect of individual attributes that contributed to the pathogenicity prediction can be viewed using the SHAP values of each attribute^17^.

The ACMG scoring scheme is rather strict when it comes to classifying CNVs. We observed that the majority of CNVs were classified as VUS. On the other hand, ISV, the machine-learning tool, was able to classify many more CNVs, however, at the cost of producing more false predictions. Evaluation of the advantages of both approaches led us to propose merging the CNV clinical impact prediction tools (ISV) with the current scoring system of the ACMG and ClinGen standards (MarCNV). In the presented work, we used selected test sets of CNVs from the ClinVar database to evaluate and compare each approach (MarCNV and ISV) to a combined method (MarCNV + ISV). We provide evidence that by employing such a combined approach, we can increase the *accuracy* of predictions as well as the number of informative (falling either into the B or P category ) CNVs compared to using the ACMG criteria only. In this way, a more precise evaluation of CNVs’ clinical impact can be achieved.

The *Accuracy* of our combined approach depends on the MarCNV score and the ISV ratio (*r*) setting. In general, the higher the ISV ratio, the greater the influence of ISV on the final prediction. For example, when *r* = 1, ISV alone will not have the power to decide the CNV outcome where the ACMG score is between -0.48 and 0.48.

Our combined approach was also verified on the samples from a clinical laboratory and confirmed that evaluation according to the ACMG criteria (MarCNV) is quite conservative (compared to CI). The combination of both approaches (including ISV) reduced the number of *uncertain predictions* and increased the number of CNVs assigned to the category P or B. However, the combined approach may also lead to some false predictions. It should be noted that CI also includes patient and family anamnesis, while automated tools such as MarCNV and ISV use only CNV coordinates as input. However, after applying the combined approach, if necessary, clinicians can still contribute significantly by evaluation of the patient's anamnesis and including their data.

In conclusion, the combined application of the ACMG scoring scheme (MarCNV) and a machine learning-based tool (ISV) leads to more accurate predictions. Furthermore, a well-established tool and a sufficiently tested machine-learning model could contribute to more reliable outcomes for predictions of CNV pathogenicity. This approach may provide valuable evidence supporting the decision-making during the evaluation and classification of CNVs, especially in clinical settings. The current ACMG standards and guidelines for interpretation of sequence variants (especially of those developed for SNVs) do consider in silico predictions as supporting evidence, although with a low weight. However, machine-learning tools for CNV evaluation, such as ISV, were developed almost simultaneously with the proposal of the ACMG standards for CNVs, and thus they could not have been inserted into the ACMG criteria scheme previously. Since there has been significant progress in such in silico prediction tools recently, we suggest they could even be included as supporting evidence with more power/weight in the future revisions of the ACMG scheme.

## Methods

### MarCNV

MarCNV is a new automated tool for the evaluation of parts of ACMG criteria^4^ (Tables 1, 2). The user is required to provide only information about the target CNV: location in hg38 reference (chromosome, start, and end) and its character (gain or loss). MarCNV then evaluates complete Sections 1 and 3, almost complete Section 2, and option 4O from Section 4 of the ACMG criteria. It is similar to the already published ClassifyCNV^6^ and AutoCNV^7^, which, however, were not available at the time of the implementation.

### ISV

ISV (Interpretation of Structural Variation) is a machine learning-based approach in which the pathogenicity of a candidate CNV is assessed by observing the counts of overlapped subcategories of genes and regulatory elements^9^. ISV is based on boosted trees, and it classifies most variants reliably and with high accuracy (~ 98%) among the compared tools^9^.

### CNV data sets

For the evaluation and training of hybrid models, we collected benign and pathogenic CNVs from the ClinVar database^18^ (downloaded on April 27th, 2021). We extracted unique copy number loss and copy number gain variants with inner coordinates lifted to GRCh38 using UCSC LiftOver tool^19^. We filtered out all CNVs shorter than 1 Kbp and longer than 5 Mbp. We included only single-copy events denoted by a multiplicity of 1 and 3 for losses and gains, respectively. The selected CNV records were then randomly divided into three disjunct data sets: *Training*-70% (loss: 8533, gain: 6589), *Validation*-15% (loss: 1829, gain: 1411) and *Testing basic*—15% (loss: 1829, gain: 1412). Training and validation sets were used only for training of the ISV model and for choosing the database of benign CNVs for MarCNV.

We also prepared two additional test sets using CNVs with more significant impact: *Testing* > 5 Mbp, including CNVs longer than 5 Mbp (loss: 1859, gain: 1328); and *Testing multiple*, including CNVs with different multiplicities (multiplicities 0 for loss and 4 for gain) (loss: 2244, gain: 472). Plots and tables presented in the Results section come from the concatenation of the following three datasets: *Testing basic, Testing* > 5 Mbp*, and Testing multiple*. The referred datasets are the same asthose used by ISV^9^.

### Testing the effect of database selection on the CNV evaluation precision

Automatic evaluation tools search for an overlap of the tested CNV in the selected databases that collect common CNVs from large-scale sequencing studies. We used MarCNV to test whether the selection of different benign CNV databases and the settings used during selection can influence the result of automated CNV evaluation. Namely, we compared evaluation outputs of the Sections 2F–G for loss and 2C-2G for gain obtained using two databases of known benign CNVs: GnomAD database^14^ and Database of Genomic Variants of all benign CNVs (DGV)^15^. Three versions of DGV databases were compared: “DGV”, which represents all CNVs in the DGV database; “DGV-GOLD”, which stands for “Gold Standard” and represents curated CNVs with INNER or OUTER CNV coordinates. In addition, analyses were performed at different population frequency thresholds, with the assumption that more common CNVs are less harmful. Namely, each of the databases was filtered to screen out the CNVs with less than 0.01%, 0.1%, 0.5%, 1%, 2%, or 10% frequency, respectively. We thus obtained 28 different databases/settings of benign CNVs for evaluation and comparison. We then calculated the *accuracy* of the prediction and the proportion of *unambiguous* CNVs, as described in the following paragraph.

### Calculation of the “accuracy” and “unambiguous” parameters used in figures

*Accuracy* was calculated as the number of correctly predicted CNVs divided by the sum of correctly and incorrectly predicted ones (considering ClinVar evaluation as the *ground truth*). The “unambiguous” parameter means the percentage of the decided/evaluated CNVs, i.e., those predicted either as B or P. Since classes LP and LB only indicate definitive classification without sufficient conclusive evidence to classify as P or B, they are represented as *uncertain predictions* in our analysis. Only CNVs with score > 0.99 and < − 0.99 were considered P and B, respectively.

### Improvement of the ACMG criteria

In order to evaluate the effect of other parameters on the result of the CNV evaluation, we tried to tweak the current ACMG criteria by changing some settings of the database search: (1) according to the ACMG criteria, both benign losses and gains are included in the database overlap search for Section 2F—we performed an analysis with settings that included only benign losses (which is more intuitive, since CNV losses are evaluated); (2) according to the ACMG criteria, only HI and TS genes with score 3 are included in the search for Sections 2A and 2B—we tested how the model performs if HI/TS genes with scores 1 and 2 are included.

### A combined approach that uses MarCNV and ISV

The joint combined MarCNV + ISV approach model can be easily represented by the following equation: *f*_*joint*_ = *ACMG*_*score*_ + *r* * (*ISV*_*probability*_ − 0.50), where *ACMG*_*score*_ is the score from an ACMG scheme (e.g., MarCNV), and *ISV*_*probability*_ is the probability returned by the ISV model (ranges from zero to one). Subtracting 0.5 from the ISV probability will decrease the final score for benign CNVs and increase the score for predicted pathogenic CNVs by 0.50 at most. Calculating the score in this way preserves the ranges set by ACMG.

The coefficient (ISV ratio) scales the contribution of ISV on the final prediction. If set to zero, the *r* predictor will be identical to the provided ACMG scheme, and raising it will increase the influence of ISV. Effect of the increasing ISV ratio from 0 to 3 on the CNV evaluation in the combined approach was tested. For *r* = 3, the ISV probability (minus 0.50 to achieve negative values for benign predictions) would be scaled by a factor of 3.

## Supplementary Information

Supplementary Figures.

Supplementary Information.

Supplementary Table S1.

Supplementary Table S2.

## Data Availability

The raw and processed data required to reproduce the above findings are available to download from https://github.com/tsladecek/cnv_interpretation/tree/master/data.

## Code availability

Scripts for reproducing results are available at https://github.com/tsladecek/cnv_interpretation. CNV evaluation using MarCNV only, using ISV only, and using combination of MarCNV + ISV (combined approach) is available at https://predict.genovisio.com/ for non-commercial use.
