# Supplementary material for: Combination of expert guidelines-based and machine learning-based approaches leads to superior accuracy of automated prediction of clinical effect of copy number variations
Source: Sci Rep. 2023 Jun 29;13:10531. doi: 10.1038/s41598-023-37352-1 (PMC10310736; doi:10.1038/s41598-023-37352-1)

Combination of Expert Guidelines-based and Machine Learning-based Approaches Leads to Superior Accuracy of Automated Prediction of Clinical Effect of Copy Number Variations

Tomáš Sládeček, Michaela Gažiová, Marcel Kucharík, Andrea Zaťková, Zuzana Pös, Ondrej Pös, Werner Krampl, Erika Tomková, Michaela Hýblová, Gabriel Minárik, Ján Radvánszky, Jaroslav Budiš, Tomáš Szemes

Supplementary Figures

Supplementary Figure S1. Comparison of ClassifyCNV, MarCNV, ISV, ClassifyCNV + ISV, and MarCNV + ISV methods of benign CNVs evaluation. The accuracy (Acc) indicates the proportion of correctly evaluated benign CNVs, whereas unambiguous (Una) represents the percentage of predicted CNVs falling into the B category. Upper panel (DEL) corresponds to losses and lower panel (DUP) corresponds to gains. The x-axis represents the number of CNVs. TN=true negative, FP=false positive, where "positive" predictions correspond with pathogenic and "negative" with benign predictions.

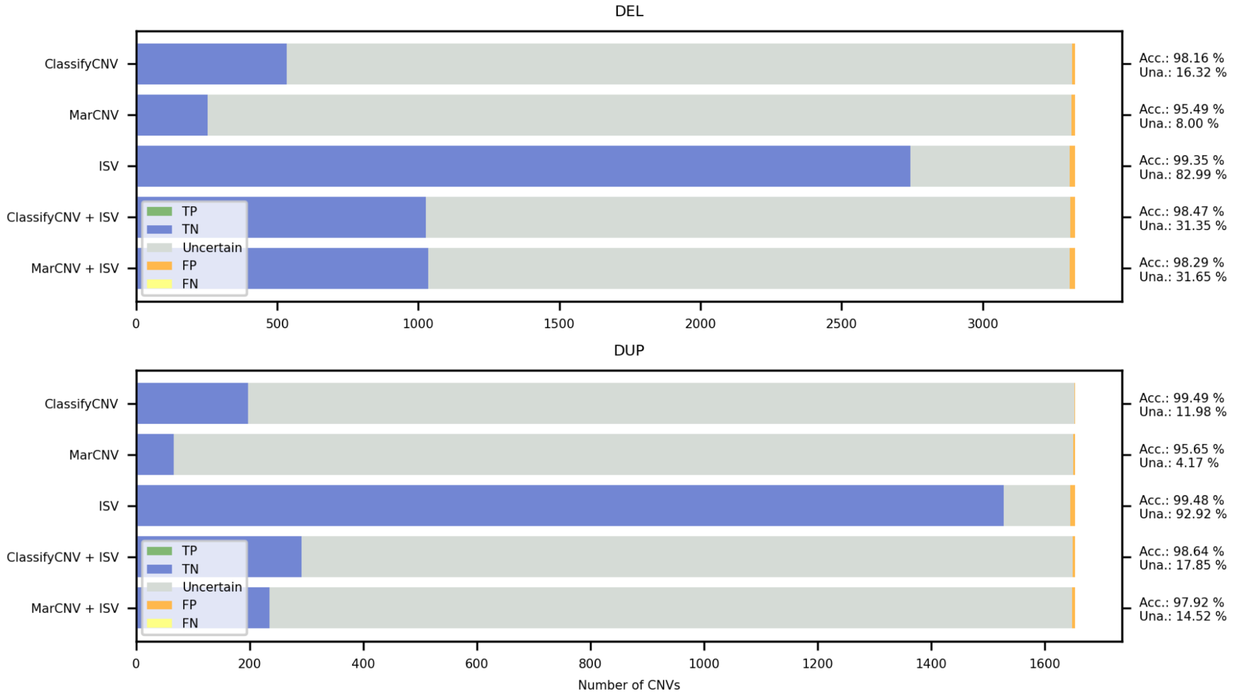

Supplementary Figure S2. Comparison of ClassifyCNV, MarCNV, ISV, ClassifyCNV + ISV, and MarCNV + ISV methods of pathogenic CNVs evaluation. The accuracy (Acc) indicates the proportion of correctly evaluated pathogenic CNVs, whereas unambiguous (Una) represents the percentage of predicted CNVs falling into the category. Upper panel (DEL) corresponds to losses and lower panel (DUP) corresponds to gains. The x-axis represents the number of CNVs. TP=true positive, FN=false negative, where "positive" predictions correspond with pathogenic and "negative" with benign predictions.

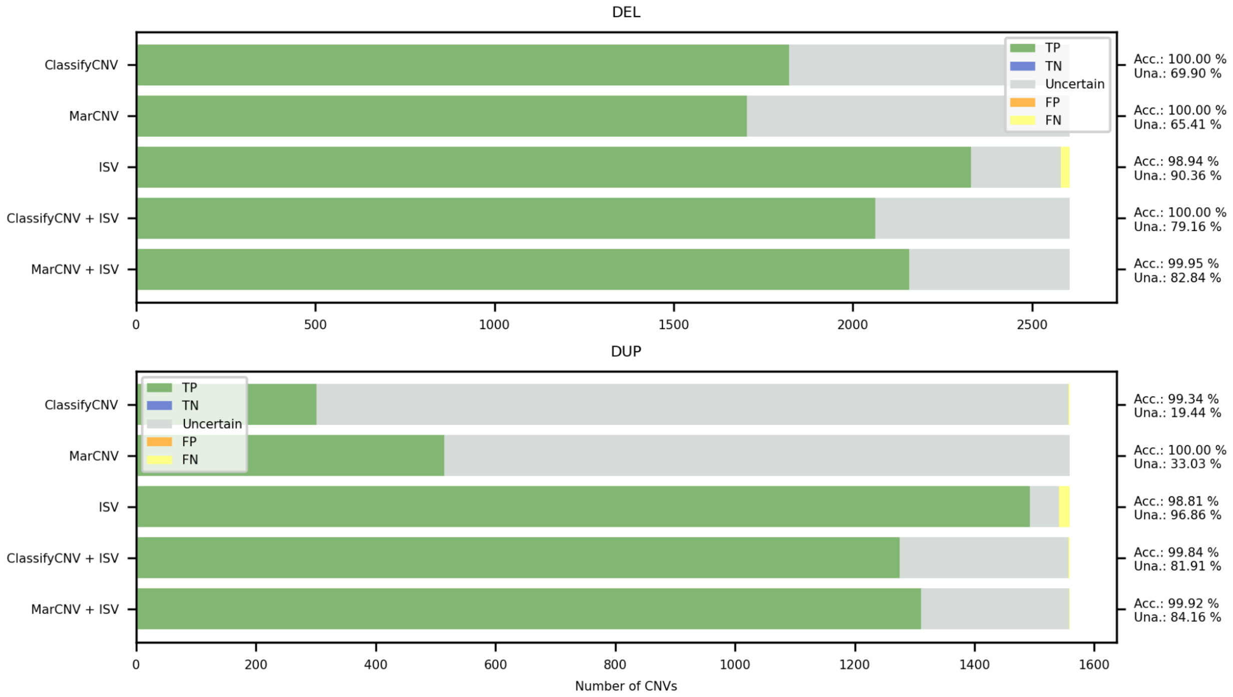

Supplementary Figure S3. Comparison of clinical significance of the CNVs test set from the ClinVar database (left side) to the evaluation obtained by the combined approach proposed in this paper (MarCNV + ISV) (right side). Test set includes *Testing basic, Testing >5MBp, and Testing multiple* datasets. Line thickness illustrates counts of CNVs, The numbers of CNVs assigned to individual categories are shown. B=benign, LB=likely benign, VUS=variant of uncertain significance, LP=likely pathogenic, and P=pathogenic. Figure was created using the SankeyMATIC tool [16].

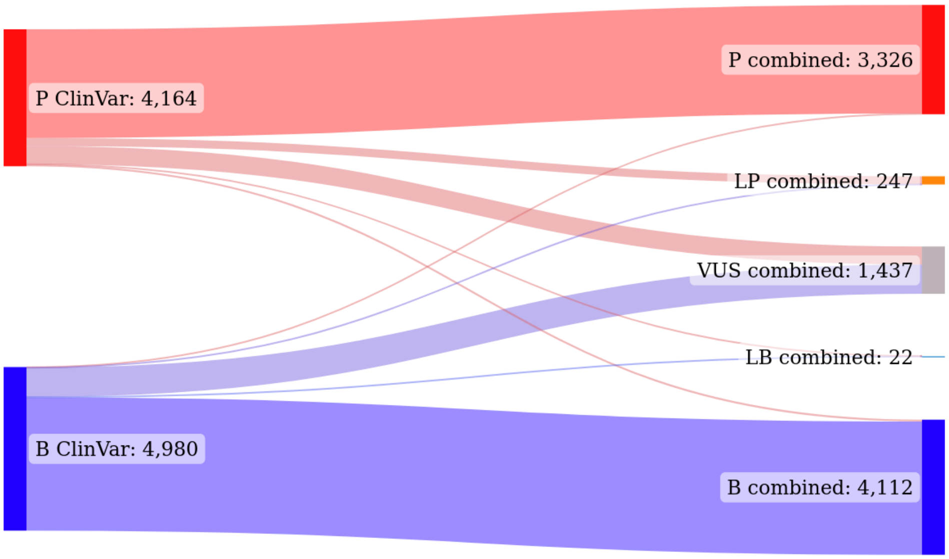

Supplementary Figures

Supplementary Figure S4 a, b: Change in the number of CNVs (obtained from the clinical laboratory) assigned to individual categories using clinical interpretation (CI) and the combined approach (MarCNV + ISV), at ISV ratios set to 0.19 (panel a) and 1.99 (panel b). As described in the main text (section 'Use of a new combined approach can increase accuracy'), these are two ratios, at which a reduction in the number of CNVs classified as VUS occurs (see also Figure 4 in the main text). The observed distinction between ISV ratios arises in the increased prediction of CNVs as VUS when using an ISV ratio of r = 0.19, which is caused by favoring scores based on ACMG criteria over ISV scores. Line thickness illustrates counts of CNVs. The numbers of CNVs assigned to individual categories are shown. B=benign, LB=likely benign, VUS=variant of uncertain significance, LP=likely pathogenic, and P=pathogenic. Figure was created using the SankeyMATIC tool [16].

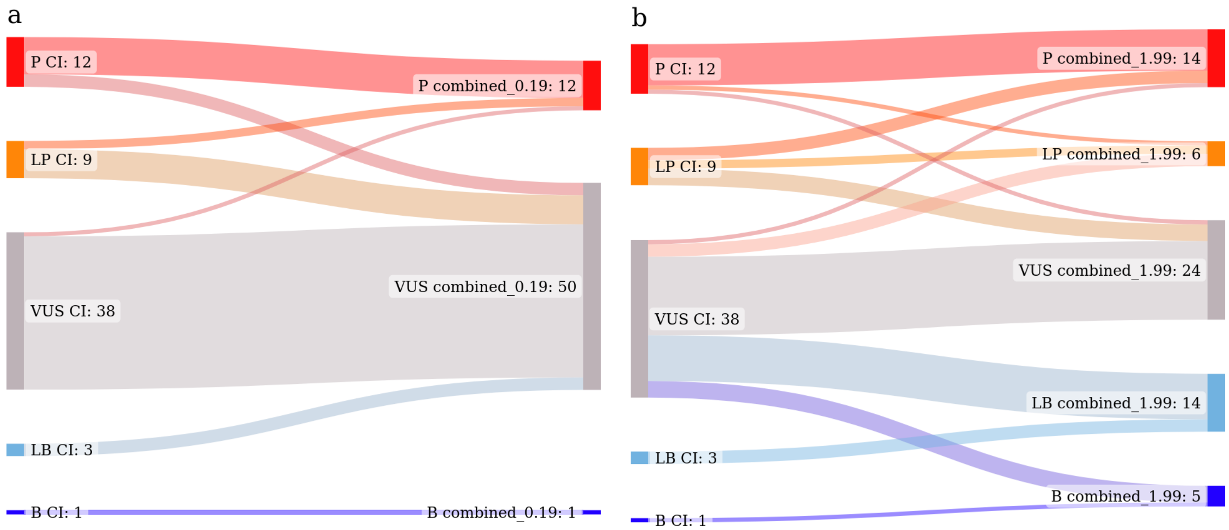

Supplementary Figure S5. Comparison of the number of CNVs (obtained from the clinical laboratory) assigned to individual categories by MarCNV and ISV tools. Line thickness illustrates counts of CNVs. The numbers of CNVs assigned to individual categories are shown. B=benign, LB=likely benign, VUS=variant of uncertain significance, LP=likely pathogenic, and P=pathogenic. Figure was created using the SankeyMATIC tool [16].

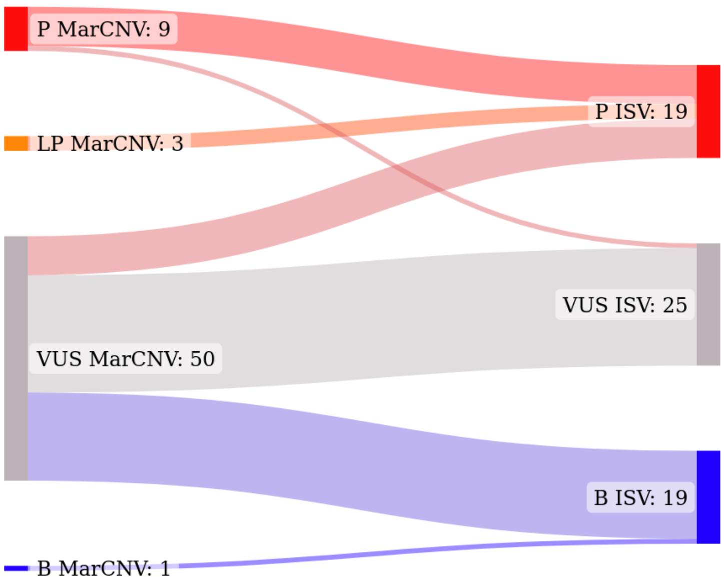

Supplement: Supplementary file 1 — Supplementary Figures. [file 41598_2023_37352_MOESM1_ESM.pdf]
