# Supplementary material for: Combination of expert guidelines-based and machine learning-based approaches leads to superior accuracy of automated prediction of clinical effect of copy number variations
Source: Sci Rep. 2023 Jun 29;13:10531. doi: 10.1038/s41598-023-37352-1 (PMC10310736; doi:10.1038/s41598-023-37352-1)
Supplement: Supplementary file 3 — Supplementary Table S1. [file 41598_2023_37352_MOESM3_ESM.pdf]

Supplementary Table S1. Pairwise comparison of classification methods with McNemar-Bowker test. The test statistic was calculated from a 3x3 contingency matrix , since there are 3 possible classification outcomes - “Benign”, “Uncertain significance”, “Pathogenic”. For ACMG based methods (MarCNV and ClassifyCNV), predictions falling to the “Likely benign” or “Likely pathogenic” categories were treated as variants of “Uncertain significance”

CNV type	Method 1	Method 2	Test Statistic	p-value
DEL	ISV	ClassifyCNV	2602.36	0
DEL	ISV	MarCNV	3063.44	0
DEL	ISV	ClassifyCNV + ISV	1839.69	0
DEL	ISV	MarCNV + ISV	1788.97	0
DEL	ClassifyCNV	MarCNV	218.73	0
DEL	ClassifyCNV	ClassifyCNV + ISV	723.45	0
DEL	ClassifyCNV	MarCNV + ISV	550.43	0
DEL	MarCNV	ClassifyCNV + ISV	897.89	0
DEL	MarCNV	MarCNV + ISV	1231.21	0
DEL	ClassifyCNV + ISV	MarCNV + ISV	39.86	$1.14 * 10^{-8}$
DUP	ISV	ClassifyCNV	2514.58	0
DUP	ISV	MarCNV	2430.01	0
DUP	ISV	ClassifyCNV + ISV	1432.18	0
DUP	ISV	MarCNV + ISV	1457.81	0
DUP	ClassifyCNV	MarCNV	299	0
DUP	ClassifyCNV	ClassifyCNV + ISV	1065.04	0
DUP	ClassifyCNV	MarCNV + ISV	1010.17	0
DUP	MarCNV	ClassifyCNV + ISV	893.96	0
DUP	MarCNV	MarCNV + ISV	960.08	0
DUP	ClassifyCNV + ISV	MarCNV + ISV	46.03	$5.58 * 10^{-10}$
