# Supplementary material for: Combination of expert guidelines-based and machine learning-based approaches leads to superior accuracy of automated prediction of clinical effect of copy number variations
Source: Sci Rep. 2023 Jun 29;13:10531. doi: 10.1038/s41598-023-37352-1 (PMC10310736; doi:10.1038/s41598-023-37352-1)
Supplement: Supplementary file 4 — Supplementary Table S2. [file 41598_2023_37352_MOESM4_ESM.pdf]

CNVs	Coordinates (GRCh38)	Clinical	MarCNV	ISV	Combined	Combined	Combined
loss	chr16:15398460-17918604	Pathogenic	Pathogenic	Pathogenic	Pathogenic	Pathogenic	Pathogenic
loss	chr17:6998753-7343741	Pathogenic	VUS	VUS	VUS	VUS	VUS
loss	chr20:25360164-25417189	VUS	VUS	VUS	VUS	VUS	VUS
loss	chr2:107988278-109669677	VUS	VUS	Pathogenic	VUS	Likely pathogenic	VUS
loss	chr3:166404111-169020566	Pathogenic	VUS	Pathogenic	VUS	Likely pathogenic	VUS
loss	chr6:26428695-27912545	VUS	VUS	VUS	VUS	VUS	VUS
gain	chr1:145687796-145802744	VUS	VUS	Benign	VUS	Benign	VUS
gain	chr2:99390192-101050636	VUS	VUS	Pathogenic	VUS	Likely pathogenic	VUS
gain	chr5:109344299-123484306	Pathogenic	Likely pathogenic	Pathogenic	Pathogenic	Pathogenic	Pathogenic
loss	chr15:22787972-23088545	Likely Pathogenic	VUS	VUS	VUS	VUS	VUS
loss	chr15:55991010-56776071	VUS	VUS	VUS	VUS	VUS	VUS
loss	chr16:21588366-21940117	VUS	VUS	VUS	VUS	VUS	VUS
loss	chr1:174371028-174836898	VUS	VUS	VUS	VUS	VUS	VUS
loss	chr7:105375646-105555122	VUS	VUS	VUS	VUS	VUS	VUS
loss	chr9:110423845-110730249	VUS	VUS	VUS	VUS	VUS	VUS
loss	chrX:16129094-16396570	VUS	VUS	Benign	VUS	Likely benign	VUS
gain	chr13:19632400-19782884	VUS	VUS	Benign	VUS	Benign	VUS
gain	chr15:31729530-32218662	VUS	VUS	VUS	VUS	VUS	VUS
gain	chr2:32470151-33024069	Likely benign	VUS	Benign	VUS	Likely benign	VUS
gain	chr6:167970736-168180532	Benign	Benign	Benign	Benign	Benign	Benign
gain	chr8:31630487-32728505	VUS	VUS	VUS	VUS	VUS	VUS
loss	chr17:1196706-1556706	Likely pathogenic	VUS	VUS	VUS	VUS	VUS
loss	chr15:22770714-28174854	Pathogenic	Pathogenic	Pathogenic	Pathogenic	Pathogenic	Pathogenic
loss	chr16:29648679-30168679	Pathogenic	VUS	Pathogenic	Likely pathogenic	Pathogenic	VUS
loss	chr22:50161571-50281571	VUS	VUS	VUS	VUS	VUS	VUS
loss	chr4:80107-3678273	Pathogenic	Likely pathogenic	Pathogenic	Pathogenic	Pathogenic	Pathogenic
loss	chr7:110719944-117039946	Pathogenic	Pathogenic	Pathogenic	Pathogenic	Pathogenic	Pathogenic
loss	chr7:152122915-159307311	Pathogenic	Pathogenic	Pathogenic	Pathogenic	Pathogenic	Pathogenic
gain	chr12:113862195-114062195	VUS	VUS	Benign	VUS	Benign	VUS
gain	chr15:30867797-32127799	VUS	VUS	Pathogenic	VUS	Likely pathogenic	VUS
gain	chr15:99239795-99739795	VUS	VUS	Benign	VUS	Likely benign	VUS
gain	chr1:147068456-148247729	Pathogenic	Pathogenic	Pathogenic	Pathogenic	Pathogenic	Pathogenic
gain	chr22:20385710-21565711	Likely pathogenic	VUS	Pathogenic	VUS	Likely pathogenic	VUS
gain	chr22:20425711-21085711	Likely pathogenic	VUS	VUS	VUS	VUS	VUS

**gain**	chr5:126304308-126864308	VUS	Pathogenic	VUS	Pathogenic	Pathogenic	Pathogenic
**gain**	chrX:26661883-32641883	Likely pathogenic	Pathogenic	Pathogenic	Pathogenic	Pathogenic	Pathogenic
**loss**	chr13:79845865-80065865	VUS	VUS	Benign	VUS	Likely benign	VUS
**loss**	chr13:79845865-80085865	VUS	VUS	Benign	VUS	Likely benign	VUS
**loss**	chr15:55887802-56767802	VUS	VUS	VUS	VUS	VUS	VUS
**loss**	chr17:796760-1776706	Likely pathogenic	VUS	Pathogenic	VUS	Pathogenic	VUS
**loss**	chr18:7840002-8300002	VUS	VUS	VUS	VUS	VUS	VUS
**loss**	chr18:67372763-68372763	VUS	VUS	VUS	VUS	VUS	VUS
**loss**	chr18:67372763-68332763	VUS	VUS	VUS	VUS	VUS	VUS
**loss**	chr22:33564014-33804013	VUS	VUS	VUS	VUS	VUS	VUS
**loss**	chr6:135418862-135638862	VUS	VUS	VUS	VUS	VUS	VUS
**loss**	chr7:111199944-111619944	Likely benign	VUS	Benign	VUS	Likely benign	VUS
**loss**	chr7:111259944-111539944	Likely benign	VUS	Benign	VUS	Likely benign	VUS
**gain**	chr15:22813068-23073068	VUS	VUS	VUS	VUS	VUS	VUS
**gain**	chr15:31727797-32127799	VUS	VUS	Benign	VUS	Likely benign	VUS
**gain**	chr15:100699795-101399795	VUS	VUS	VUS	VUS	VUS	VUS
**gain**	chr16:15026143-16166143	Likely pathogenic	VUS	Pathogenic	VUS	Likely pathogenic	VUS
**gain**	chr22:21945628-22205609	VUS	VUS	Benign	VUS	Likely benign	VUS
**gain**	chr5:81284181-81804181	VUS	VUS	Benign	VUS	Likely benign	VUS
**gain**	chr6:156618866-156878866	VUS	VUS	Benign	VUS	Likely benign	VUS
**gain**	chr6:162278968-162478968	VUS	VUS	Benign	VUS	Likely benign	VUS
**gain**	chrX:7661959-8431959	VUS	VUS	Benign	VUS	Likely benign	VUS
**loss**	chr2:50872862-51452862	Likely pathogenic	Likely pathogenic	Pathogenic	Pathogenic	Pathogenic	Pathogenic
**gain**	chr3:163602212-198033129	Pathogenic	Pathogenic	Pathogenic	Pathogenic	Pathogenic	Pathogenic
**gain**	chr19:49896743-50156743	VUS	VUS	Benign	VUS	Likely benign	VUS
**gain**	chr17:59722639-72763861	Pathogenic	Pathogenic	Pathogenic	Pathogenic	Pathogenic	Pathogenic
**loss**	chr15:22813068-23073068	Likely pathogenic	VUS	VUS	VUS	VUS	VUS
**gain**	chr15:89496769-89736769	VUS	VUS	VUS	VUS	VUS	VUS
**gain**	chr1:175450864-175770864	VUS	VUS	Benign	VUS	Benign	VUS

Supplementary Table S2: Summary of monoallelic CNVs identified in 63 patients from clinical laboratory. CNV type and coordinates are shown, as well as original Clinical Interpretation (CI), together with interpretations performed by MarCNV, ISV and the combined approach. "*r*" represents the ISV ratio set during the evaluation. *r* =1 is the default ISV ratio.
